# The second molecular epidemiological study of HIV infection in Mongolia between 2010 and 2016

**DOI:** 10.1371/journal.pone.0189605

**Published:** 2017-12-15

**Authors:** Davaalkham Jagdagsuren, Tsunefusa Hayashida, Misao Takano, Erdenetuya Gombo, Setsen Zayasaikhan, Naomi Kanayama, Kiyoto Tsuchiya, Shinichi Oka

**Affiliations:** 1 AIDS/STI Surveillance and Research Department, National Center for Communicable Diseases, Ulaanbaatar, Mongolia; 2 AIDS Clinical Center, National Center for Global Health and Medicine, Tokyo, Japan; 3 Center for AIDS Research, Kumamoto University, Kumamoto, Japan; University of Malaya, MALAYSIA

## Abstract

**Objective:**

Our previous 2005–2009 molecular epidemiological study in Mongolia identified a hot spot of HIV-1 transmission in men who have sex with men (MSM). To control the infection, we collaborated with NGOs to promote safer sex and HIV testing since mid-2010. In this study, we carried out the second molecular epidemiological survey between 2010 and 2016 to determine the status of HIV-1 infection in Mongolia.

**Methods:**

The study included 143 new cases of HIV-1 infection. Viral RNA was extracted from stocked plasma samples and sequenced for the *pol* and the *env* regions using the Sanger method. Near-full length sequencing using MiSeq was performed in 3 patients who were suspected to be infected with recombinant HIV-1. Phylogenetic analysis was performed using the neighbor-joining method and Bayesian Markov chain Monte Carlo method.

**Results:**

MSM was the main transmission route in the previous and current studies. However, heterosexual route showed a significant increase in recent years. Phylogenetic analysis documented three taxa; Mongolian B, Korean B, and CRF51_01B, though the former two were also observed in the previous study. CRF51_01B, which originated from Singapore and Malaysia, was confirmed by near-full length sequencing. Although these strains were mainly detected in MSM, they were also found in increasing numbers of heterosexual males and females. Bayesian phylogenetic analysis estimated transmission of CRF51_01B into Mongolia around early 2000s. An extended Bayesian skyline plot showed a rapid increase in the effective population size of Mongolian B cluster around 2004 and that of CRF51_01B cluster around 2011.

**Conclusions:**

HIV-1 infection might expand to the general population in Mongolia. Our study documented a new cluster of HIV-1 transmission, enhancing our understanding of the epidemiological status of HIV-1 in Mongolia.

## Introduction

Mongolia is a low HIV-1 prevalence country. According to the national surveillance data, there was virtually no HIV-1 infected individuals in 1990s, with only 5 cases reported from 1992 to 2004. However, the number of newly diagnosed cases have increased exponentially since 2005. The reported cumulative number was 225 cases for the period of 1992–2016 [1]. Among the total population of Mongolia of 3 million people, the number of individuals living with HIV-1 was still less than 600 cases, as estimated in early 2017 (according to HIV Spectrum Estimation /Spectrum V5.7, AIDS/Sexually Transmitted Infection (STI) Surveillance and Research Department, National Center for Communicable Diseases (NCCD); National Consultation Meeting on HIV Estimation and Global AIDS Monitoring, 17 March 2017, Ulaanbaatar, Mongolia). However, the surveillance documented a steep rise of HIV-1 epidemic among men who have sex with men (MSM). According to the HIV/STI Surveillance Survey Report 2014, the prevalence of HIV-1 in MSM in urban settings reached 13.7 percent (adjusted prevalence– 12%), indicating surprisingly rapid expansion of HIV-1 infection among MSM in this decade.

The prevalence of STIs, which increase the risk of HIV transmission [2], has grown in Mongolia not only in at-risk populations, but also in the general public. In particular, prevalence of syphilis increased in all population groups including MSM, female sex workers, reproductive-age men and women, and pregnant women in this decade. Positive syphilis serology in pregnant women reached 5.2% (HIV/STI Surveillance Survey Report 2014), which was almost three times higher than the global target of less than 2%.

This rapid expansion of HIV-1 infection and high prevalence of STIs should raise concern and effective approaches to control further expansion of HIV-1 infection are needed. Our previous molecular epidemiological analysis of HIV-1 infection in Mongolia between 2005 and 2009 found a genetically close cluster composed of MSM, and concluded that the cluster was a hot spot for HIV-1 transmission in MSM [3]. The finding indicated that comprehensive preventive actions were crucial to keep the rate of HIV-1 infection low in Mongolia. This assessment prompted us to collaborate with non-government organizations (NGOs) that supported MSM by promoting safer sex and HIV testing since mid-2010. Moreover, the Mongolian Government has implemented the treat-all strategy irrespective of CD4 counts for MSM since 2013. The objectives of this study were to explore the efficacy of preventive measures in MSM and treat-all strategy for MSM, and to monitor the current status of HIV-1 infection in Mongolia by molecular epidemiological analysis.

## Materials and methods

### Subjects

This study was carried out at the AIDS/STI Surveillance and Research Department, NCCD in Mongolia. Our team conducted previously a molecular epidemiological study in 2009 [3]. The present study included 143 Mongolian HIV-1-positive patients who were diagnosed between January 2010 and December 2016. A written informed consent was obtained from the patients by the clinic personnel for withdrawal of approximately 10 ml of blood. The collected plasma samples were sent to the AIDS Clinical Center, National Center for Global Health and Medicine (NCGM), Tokyo, Japan, for further analysis. The study protocol was approved by ethics committees of NCGM (NCGM-1426) and the Ministry of Health, Mongolia (2013 #03 and 2016 #02).

### Sanger sequencing

Viral RNA was extracted from the plasma samples using QIAamp Viral RNA Mini Kit (Qiagen, Tokyo, Japan) and stored at -80°C. RT-PCR was conducted using SuperScript III Reverse Transcriptase (Thermo Fisher Scientific, Kanagawa, Japan). Subsequently, nested PCR was conducted using TaKaRa Ex Taq Hot Start Version (Takara Bio, Shiga, Japan). The primer set used in our previous study was used to amplify the *pol* (HXB2: 2243–3308) and the *env* (HXB2: 6834–7281) regions of HIV-1 [3]. The amplified DNA was purified using QIAquick PCR Purification Kit (Qiagen), then sequenced using BigDye Terminator v3.1 Cycle Sequencing Kit (Thermo Fisher Scientific) and 3730 DNA Analyzer (Thermo Fisher Scientific).

### Near-full length sequencing

The near-full length (HXB2: 706–9531) of HIV-1 genome sequencing was performed using MiSeq (Illumina, Tokyo) according to the method reported by Ode et al. [4] with minor modifications. Briefly, five segments that covered the HIV-1 genome were amplified by PCR from the extracted viral RNA described above. Instead of the third segment in the original method (HXB2: 4169–7652), we amplified 3–1 (HXB2: 4168–5979) and 3–2 (HXB2: 5803–7637) segments using another primer set. RT-PCR primers for the 3–1 segment was forward *in-env* V5 1st F (same as the primer of Ode et al. [4]) and reverse mng3-1R (5’ CTT GAT GAG TCT CAC TGT CTT GA 3’, HXB2: 6029–6007). RT-PCR primers for 3–2 segment were forward mng3-2F (5’ CCA CCC CTT CCC AGT GTT A 3’, HXB2: 5521–5539) and reverse mng3R1 (5’ CCC ATA GTG CTT CCT GCT G 3’, HXB2: 7816–7798). Nested PCR primers for 3–1 segment were forward mng3F2 (5’ GCA TGG GTA CCA GCA CAC A 3’, HXB2: 4149–4167) and reverse mng3midR (5’ TTC GTC GCT GTC TCC GCT TC 3’, HXB2: 5999–5980). Nested PCR primers for 3–2 segment were forward mng3midF (5’ TTG GGT GTC AAC ATA GCA GAA TA 3’, HXB2: 5780–5802) and reverse mng3R2 (5’ TCT CCA ATT GTC CTT TAT ATT TCC TCC 3’, HXB2: 7664–7638). PrimeScript II (Takara Bio) was used as reverse transcriptase, and PrimeStar GXL (Takara Bio) was used as DNA polymerase. The five amplified fragments were collected in a tube and purified by QIAquick PCR Purification Kit. Nextera XT Library Preparation Kit (Illumina) was used to generate the library and then pyrosequencing was performed by MiSeq.

### Data analysis

Statistical analysis was performed using IBM SPSS Statistics Version 23 (IBM Japan, Tokyo). The reference sequence for phylogenetic analyses was obtained from the Basic Local Alignment Search Tool (BLAST) of the National Center for Biotechnology Information (https://blast.ncbi.nlm.nih.gov/Blast.cgi) and the HIV database of the Los Alamos National Laboratory (https://www.hiv.lanl.gov/content/index). The sequence data were aligned by ClustalW. Phylogenetic analysis was conducted by MEGA 7.0.26 [5] using the neighbor-joining method and Kimura 2-parameter model with 1,000 bootstrap replications. A cluster was defined when the bootstrap score was ≥90%. The HIV-1 subtype and circulating recombinant form (CRF) were determined by the phylogenetic analysis described above, the Recombinant Identification Program in the HIV database of the Los Alamos National Laboratory, and SimPlot 3.5.1 [6]. The breakpoints of recombination were drawn using the Recombinant HIV-1 Drawing Tool v2.1.0 in the HIV database of the Los Alamos National Laboratory. For the data obtained from MiSeq, trimming and filtering of the sequence data was conducted by the FASTX-toolkit (http://hannonlab.cshl.edu/fastx_toolkit/index.html), *de novo* assembly was conducted by VICUNA [7] and mapping was conducted by BWA-MEM [8]. The time to the most recent common ancestor (tMRCA) was estimated by maximum clade credibility (MCC) tree analysis using the Bayesian Markov chain Monte Carlo (MCMC) method. The analysis was conducted by BEAST 2.4.3 [9] with 100,000,000 states, logged every 10,000 states, and 10% burn-in. A general time reversible model with gamma distribution and invariant sites, relaxed clock log normal model, and coalescent exponential population model were adopted because of the favorable Bayes Factor analyzed by Tracer 1.5.0 in the BEAST package. A cluster was defined when the posterior probability was ≥0.9. Extended Bayesian skyline plot analysis was performed by BEAST, then effective population size was illustrated by RStudio 1.0.44 (https://www.rstudio.com).

## Results

### Transmission route

**Fig 1:**
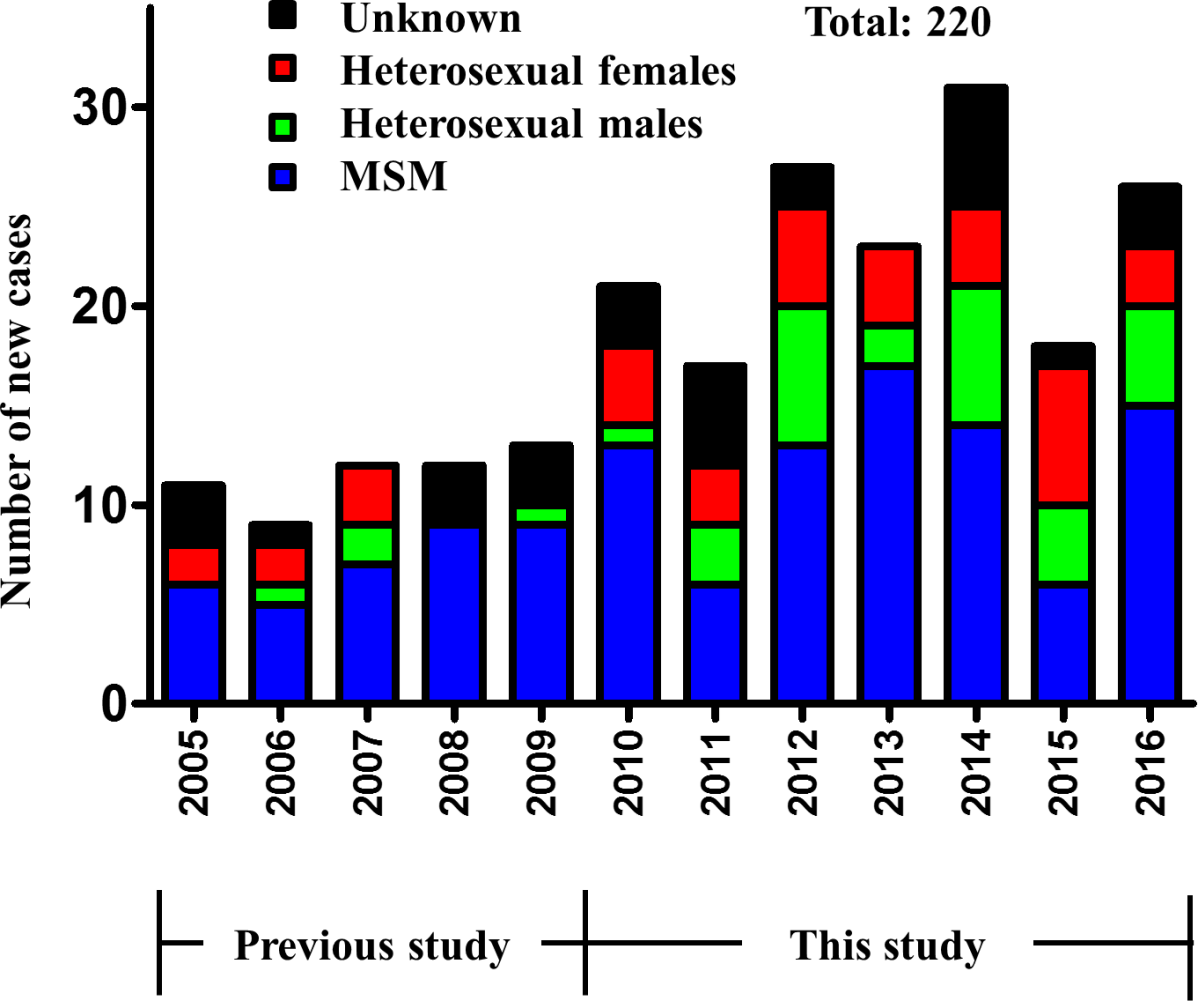
Number of newly diagnosed cases with HIV-1 infection in Mongolia, stratified by route of transmission. This graph shows 220 registered cases with HIV-1 infection in Mongolia between 2005 and 2016.

Fig 1 shows all reported cases (220) of HIV-1 infection in Mongolia during the period of 2005–2016. The present study focused on the period of 2005–2016 only, since there were only 5 cases of HIV-1 infection reported during 1992–2004 (Fig 1). The study subjects represented all those who agreed to participate in our previous 2005–2009 study (n = 47) and 143 diagnosed between 2010 and 2016. The transmission route was classified according to the risk factors in these patients. To analyze the change in transmission route, the study period was divided into the first part (2005–2010) and the second part (2011–2016). Although MSM was the main transmission route throughout the entire study period, the percentage of MSM decreased from 75.4% in the first part to 56.8% in the second part, while that of heterosexual males increased from 7.7% to 22.4%, and that of heterosexual females increased from 16.9% to 20.8%, respectively. These changes were statistically significant (*p* = 0.018, Pearson’s chi-square test, Table 1).

**Table 1:** Transmission route of HIV-1 infection in Mongolia.

Transmission route	2005–2010	(%)	2011–2016	(%)	*p*^a^
MSM	49	(75.4)	71	(56.8)	
Heterosexual males	5	(7.7)	28	(22.4)	
Heterosexual females	11	(16.9)	26	(20.8)	
Total	65		125		0.018

^a^Pearson’s chi-square test.

### Sequencing analysis

**Fig 2:**
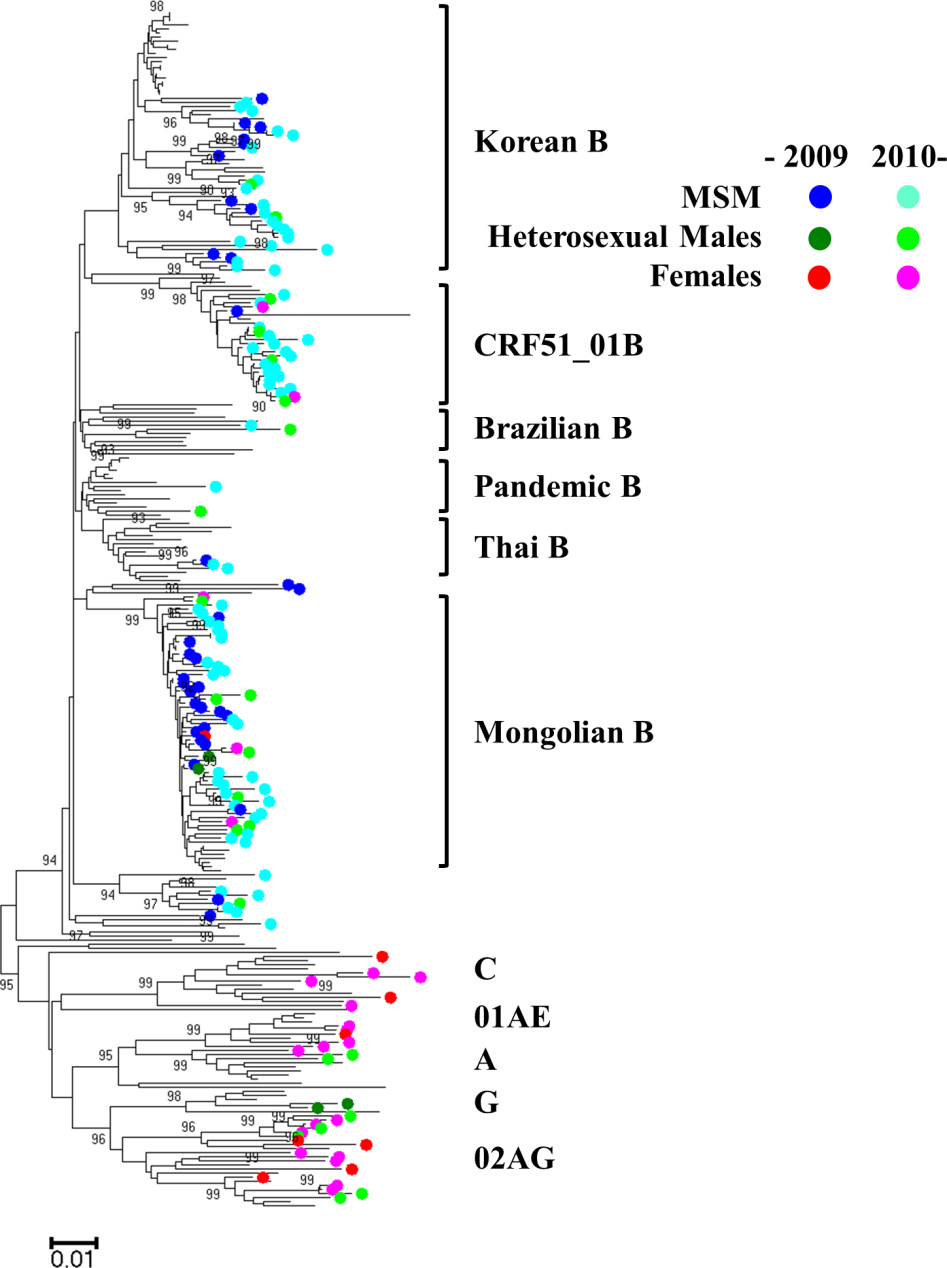
Phylogenetic analysis of the *pol* region (HXB2: 2244–3308) for HIV-1 sequences. The evolutionary history was inferred using the neighbor-joining method with the Kimura 2-parameter method. The analysis involved 293 nucleotide sequences in the *pol* region of HIV-1. MSM: men who have sex with men.

PCR was conducted on the 143 plasma samples obtained from HIV-1 positive patients. Sequencing was successful in 120 cases in the *pol* region and 118 cases in the *env* region (accession numbers: LC311885—LC312128). Specifically, both the *pol* and the *env* regions were successfully amplified in 114 samples, the *pol* region only in 6 samples, and the *env* region only in 4 samples. Phylogenetic analyses were performed on these sequences, additional sequences from our previous study (46 sequences in *pol* and 44 sequences in *env*) [3], and reference sequences obtained from the BLAST search and HIV database of the Los Alamos National Laboratory in the *pol* and the *env* regions independently (127 sequences in *pol* and 107 sequences in *env*). Three large taxa composed of Mongolian samples were observed in both the *pol* (Fig 2) and *env* regions (S1 Fig). First, the Mongolian B taxon formed significant clusters in both the *pol* and *env* regions (bootstrap value: 99 and 99, respectively), and contained samples that formed a cluster in our previous study [3]. The Mongolian B cluster also contained 8 reference sequences reported from Korea [10, 11] (accession numbers: EF157877, HQ026633, KX692417, KX692418, KX692420, KX692421, KX692422, KX692796) in the *pol* region (S2 Fig), while no similar reference sequence was found in the *env* region on the BLAST search (S3 Fig). Second, the Korean B taxon was observed in both the *pol* and the *env* regions, though the bootstrap values of the Korean B taxa were not high enough to identify these taxa as a cluster (74 in the *pol* region and 0 in the *env* region). Third, the CRF51_01B taxon was composed of subtype B in the *pol* region and CRF01_AE in the *env* region with high bootstrap values (98 and 99, respectively). The CRF51_01B had been recently reported in Singapore and Malaysia [12–15]. The near-full length sequence of this virus will be shown later. The CRF51_01B cluster was newly identified in Mongolia and was not found in the previous study [3]; however, it turned out that this cluster contained one case (08MNG4608) reported in our previous study (S4 and S5 Figs).

Table 2 shows the number of subtypes that agreed in both the *pol* and the *env* regions. The most prevalent subtype was subtype B, followed by CRF51_01B. MSM were dominant in subtype B, including Mongolian B and Korean B, and in CRF51_01B. On the other hand, heterosexual males and females were dominant in other subtypes but not in B and CRF51_01B. Interestingly, heterosexual males and females were also found among Mongolian B and CRF51_01B, but no heterosexual females were found in Korean B (Fig 2 and S1 Fig). We also divided the dominantly circulating strains of HIV-1 in Mongolia (including Mongolian B, Korean B, and CRF51_01B) from other strains (S1 Table). The rates of dominantly circulating strains were almost the same between 2005–2010 (69.6%) and 2011–2016 (69.1%). Among the dominantly circulating strains, the percentages of MSM significantly decreased from 92.3% to 71.6% (*p* = 0.013, Fisher’s exact test). Among other strains, the percentages of MSM decreased from 35.3% to 26.7%, though the latter change was not significant (*p* = 0.741, Fisher’s exact test).

**Table 2:** Subtypes of HIV-1 in Mongolia.

Subtype	1997–2009^a^	2010–2016
B	34	68
CRF51_01B	1	21
CRF02_AG	4	12
A	0	3
C	2	3
CRF01_AE	1	4
G	2	0
Not available^b^	5	32
Total	49	143

^a^Based on our previous study [3].

^b^This table shows the number of subtypes that agreed in both the *pol* and the *env* regions. Thus, missing in this table are cases with discordant results between the *pol* and the *env* regions (n = 3), success in subtyping only one region (12 cases), and failure in subtyping both regions (22 cases).

### Near-full length sequencing

**Fig 3:**
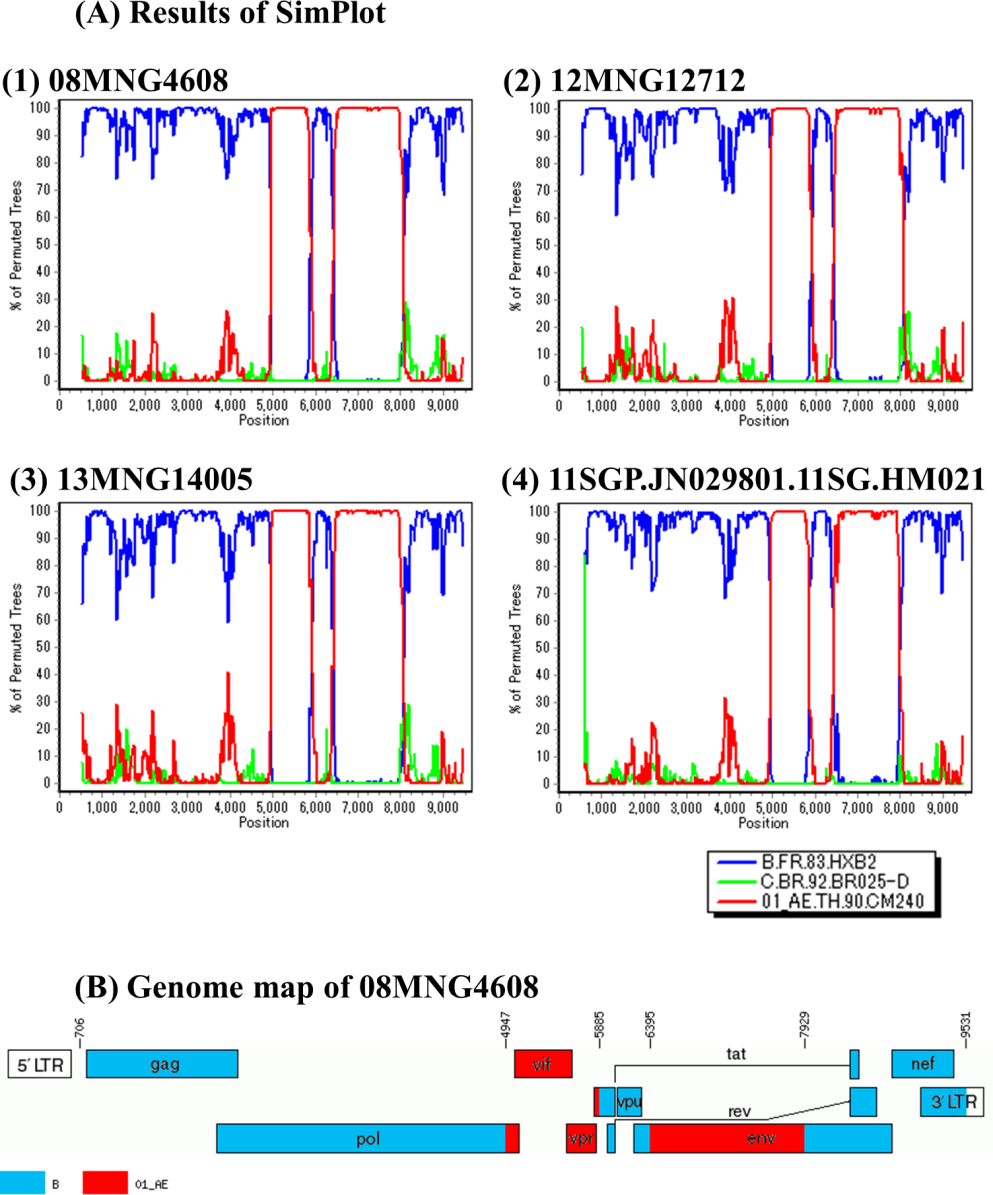
Breakpoints of recombinant HIV-1 (HXB2: 706–9531). (A) Bootscan analysis was conducted by SimPlot. Results of three Mongolian samples (08MNG4608, 12MNG12712, and 13MNG14005) and one reference sequence (11SGP.JN029801.11SG.HM021). (B) Genome map of 08MNG4608 was drawn by the Recombinant HIV-1 Drawing Tool.

To confirm that the samples in CRF51_01B cluster were definitely CRF51_01B, near-full length sequencing was performed on 3 samples in this cluster (08MNG4608, 12MNG12712, and 13MNG14005. Accession numbers: LC312713—LC312715). Near-full length sequences of these three samples and reference sequence reported as CRF51_01B (11SGP.JN029801.11SG.HM021) [12] were analyzed by SimPlot to identify the breakpoints of recombination. These breakpoints agreed completely (Fig 3). Phylogenetic analysis using near-full length of HIV-1 sequences identified a significant cluster containing these three samples and CRF51_01B reference sequence, and the CRF51_01B cluster was clearly distinguished from other CRFs composed of 01AE and B (S6 Fig). These results confirmed that these three Mongolian samples belonged to CRF51_01B.

### Estimation of tMRCA

**Fig 4:**
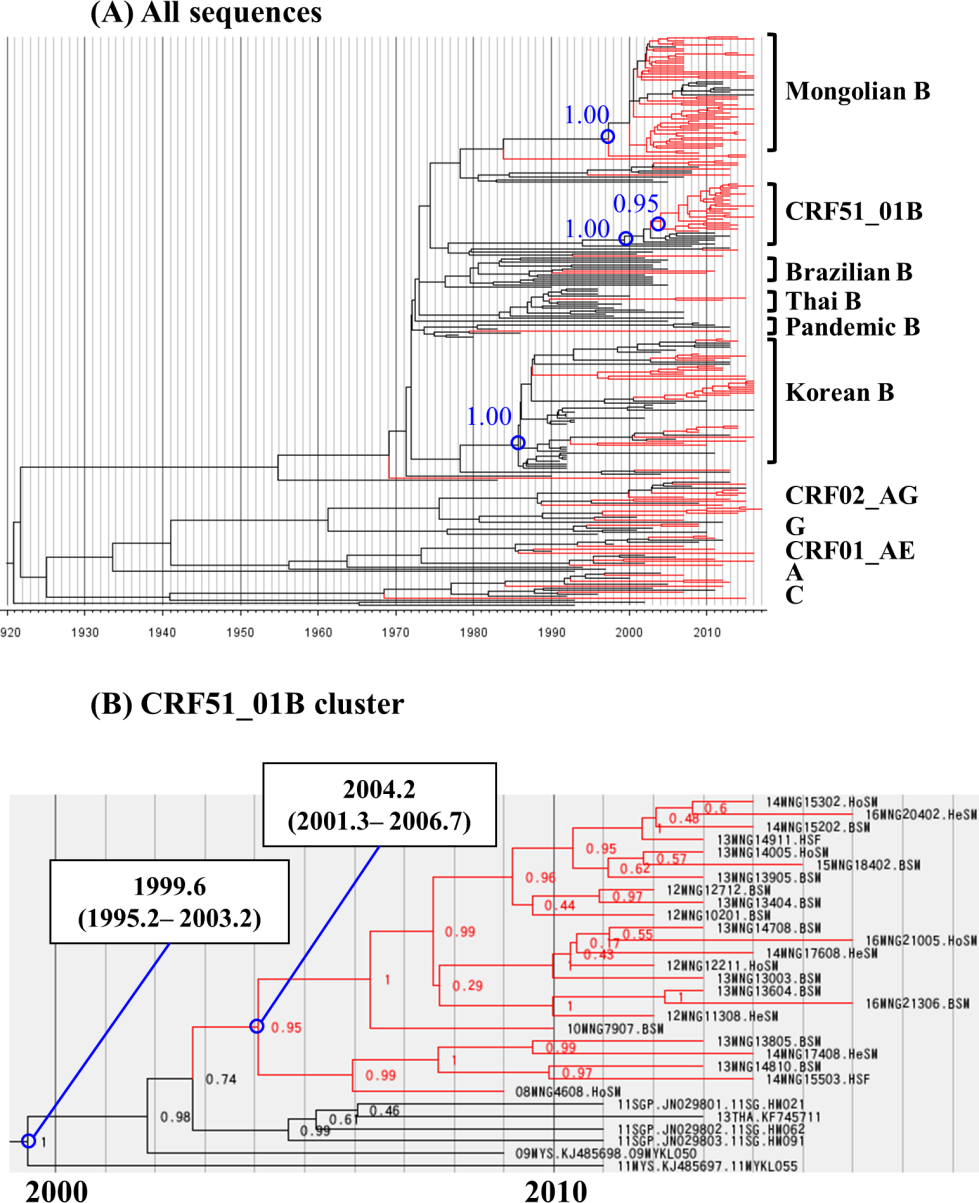
MCC tree of Bayesian MCMC analysis in the *pol* region (HXB2: 2244–3308). Chronological phylogenetic relationship in the *pol* region. The analysis involved 275 nucleotide sequences in the *pol* regions (152 Mongolian samples and 123 reference sequences). Mongolian samples are indicated by the red branches. Reference sequences are indicated by the black branches. Time scale is shown under the tree. (A) All sequences in the *pol* region. Blue numbers show posterior probability. (B) Close up of CRF51_01B cluster in the *pol* region. Blue circles: common ancestor. Data in square fields represent the median tMRCA and 95% highest posterior density interval. Numbers at the nodes represent posterior probability. MCC: maximum clade credibility. MCMC: Markov chain Monte Carlo. tMRCA: time to the most recent common ancestor.

The MCC tree analysis was performed with the sequences encoding the collection year to estimate the time of expansion of these clusters in Mongolia by the Bayesian MCMC method in the *pol* (152 Mongolian samples and 123 reference sequences, Fig 4A) and the *env* (151 Mongolian samples and 104 reference sequences, S7A Fig) regions. The median evolutionary rate per site per year was 2.15 x10^-3^ (95% highest posterior density (HPD): 1.78 x10^-3^ to 2.52 x10^-3^) in the *pol* region, and 4.58 x10^-3^ (95% HPD: 3.91 x10^-3^ to 5.28 x10^-3^) in the *env* region. Three large taxa were observed in both of the *pol* and the *env* MCC trees; Mongolian B taxon, Korean B taxon, and CRF51_01B taxon. The members of these taxa in the MCC tree analysis agreed well with that of taxa in the NJ tree analysis.

The Mongolian B cluster contained 51 and 51 sequences from Mongolia in the *pol* and the *env* regions, respectively. In the *pol* region, 8 reference sequences from Korea were located in the Mongolian B cluster. On the other hand, in the *env* region, no reference sequence was observed based on BLAST search. The median tMRCA of Mongolian B cluster was 1997.6 (95% HPD: 1992.6 to 2002.5) in the *pol* region, and 1993.1 (95% HPD: 1986.9 to 1999.2) in the *env* region.

The Korean B taxa contained 33 and 32 sequences from Mongolia, and 31 and 15 reference sequences in the *pol* and the *env* regions, respectively. The median tMRCA of Korean B cluster was 1986.4 (95% HPD: 1983.0 to 1989.2) in the *pol* region, and 1983.7 (95% HPD: 1979.1 to 1987.8) in the *env* region. Within the Korean B cluster, several common ancestors of only Mongolian samples were found in both the *pol* and the *env* MCC trees with posterior probability of >0.9. This finding indicates the independence of these common ancestors.

The CRF51_01B cluster contained 24 and 25 sequences from Mongolia, and 6 and 5 reference sequences in the *pol* (Fig 4B) and the *env* regions, respectively (S7B Fig). The median tMRCA of CRF51_01B cluster was 1999.6 (95% HPD: 1995.2 to 2003.2) in the *pol* region, and 1999.8 (95% HPD: 1995.5 to 2003.7) in the *env* region. In the CRF51_01B cluster, the Mongolian samples and other reference sequences were separated, as was applicable in both the *pol* and *env* regions. The median tMRCA of only the Mongolian samples in CRF51_01B cluster was 2004.2 (95% HPD: 2001.3 to 2006.7) in the *pol* region, and 2003.7 (95% HPD: 2001.2 to 2006.2) in the *env* region.

**Fig 5:**
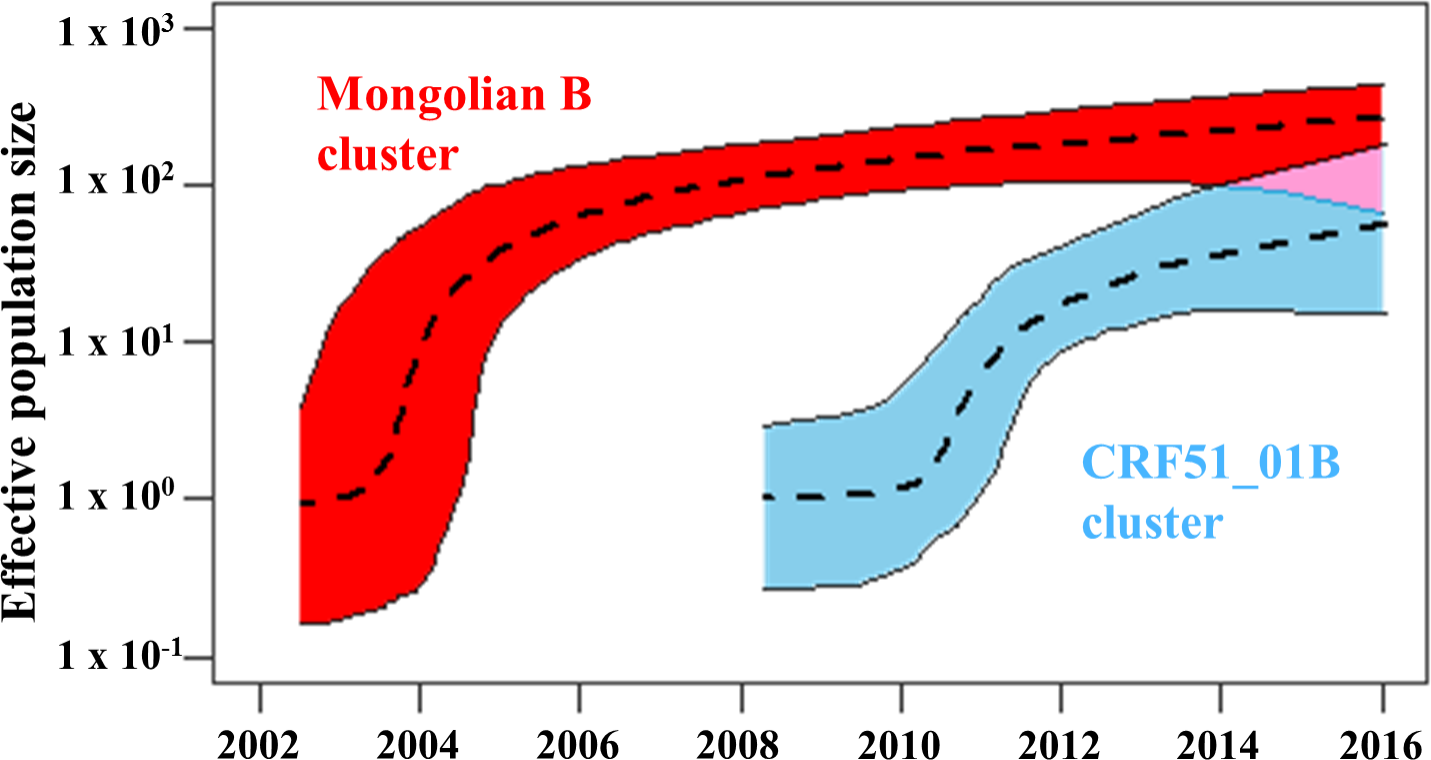
Effective population size of HIV-1 estimated by extended Bayesian skyline plot analysis based on the *pol* and the *env* sequences. Effective population size of HIV-1 in Mongolian B cluster (red) and CRF51_01B cluster (blue). The median and 95% central posterior density intervals of the effective population size are shown in log scale.

To estimate the trend in viral population size, we also conducted an extended Bayesian skyline plot analysis based on both the *pol* and the *env* sequences of Mongolian B cluster and CRF51_01B cluster, independently (Fig 5). The effective population size of Mongolian B cluster seemed to have increased steeply around 2004, followed by a gradual increase. The effective population size of CRF51_01B cluster seemed to have increased rapidly around 2011, followed by a gradual increase.

## Discussion

The aim of this study was to clarify the current molecular epidemiology of HIV-1 infection in Mongolia. Our previous study identified a large cluster in subtype B, indicating HIV-1 spread rapidly during a relatively short period with the same ancestor virus among MSM in Mongolia [3]. To limit further expansion of HIV-1 infection, various educational activities and treatment were implemented. Also, monitoring of epidemiological status was necessary to promote effective approaches. Based on phylogenetic analysis, our study identified three large taxa, which were the main circulating strains in Mongolia. Firstly, the Mongolian B cluster, which was identified in the previous study [3], seemed to continue its growth. In the NJ trees of the *pol* and the *env* regions, the samples of Mongolian B cluster in this study showed more diversity than in the previous study [3]. This is plausible since it could be affected by differences in the sampling points. The reference sequences reported from Korea were found within the Mongolian B cluster, indicating the circulation of this strain not only in Mongolia but also in Korea, though it seemed to be a minor case in Korea [11].

Secondly, the Korean B taxon was found in both the NJ and MCC tree analyses, though the bootstrap values (in the *pol* and *env* regions) and the posterior probability (in the *env* region) were not high enough regarding the taxon as a cluster. A similar low bootstrap value for the Korean B taxon by the NJ tree analysis was also reported by another group [16]. Interestingly, several common ancestors of the Mongolian samples only were identified, with high posterior probability, within the Korean B taxa, suggesting that there were several imported infection cases from Korea to Mongolia, followed by independent domestic infection in Mongolia.

Thirdly, the CRF51_01B cluster was found in Mongolian samples. The reference sequence of CRF51_01B was reported in Singapore and Malaysia [12–15]. We confirmed that three Mongolian samples in this CRF51_01B cluster were definitely CRF51_01B, using near-full length sequencing. The MCC tree analysis in both the *pol* and the *env* regions separated the Mongolian sequences from the reference sequences with high posterior probability. These findings suggest that CRF51_01B was transmitted from Singapore or Malaysia to Mongolia in the early 2000s. At that time, it seemed there was only one imported founder of CRF51_01B and this virus spread mainly among MSM in Mongolia. However, this virus has spread to heterosexual males and females, with potential further spread to the general population. The estimated tMRCA of CRF51_01B, including both Mongolian sequences and reference sequences, was 1999.5 in the pol region and 2000.2 in the env region, which was comparable to that of the other study [13].

The present study has certain limitations. First, the study was conducted using transmission risk factors, viral sequences, and annual sampling. We could not determine the traveling history of the study participants. Thus, we could not confirm the viral movement observed by molecular epidemiological analysis. Second, we also did not have information as to whether there was a difference in population composition such as age distribution and residence among Mongolian B cluster, Korean B taxa, and CRF51_01B cluster.

A decrease in effective population size would have been observed if the preventive measures and treat-all strategy for MSM had worked. In this study, neither the Mongolian B cluster nor CRF51_01B cluster decreased their effective population size nationwide (Fig 5). In this regard, the effective population size of HIV in the latest years tends to be flat in Bayesian skyline plot analysis [17]. Thus, one cannot conclude that these preventive measures failed to suppress HIV-1 expansion. There may be still many patients who do not know their HIV status. They are not treated with antiretroviral drugs, and have probably transmitted HIV-1 infection to their sexual partners. To employ preventive treatment, more HIV testing is necessary. However, with regard to the transmission route, the proportion of MSM has decreased significantly in recent years (Table 1). One possibility is that the preventive measures applied by the NGOs might have had some effects in this population, although MSM is still the major risk factor of HIV-1 infection in Mongolia. Another possibility is that dominantly circulating strain of HIV-1 might be spreading to the general population. In addition, the increase in heterosexual males and females was not because of infection by other strains, which was mainly considered as imported infection (S1 Table). Further and larger preventive measures must be applied to control the spread of infection in the future.

In conclusion, our study documented a new cluster of HIV-1 infection, indicating that the current preventive measures are inadequate. Continuous monitoring should provide a clear picture of the epidemiological status and allow us design effective strategies to control HIV-1 infection.

## Supporting information

S1 Table Comparison of HIV-1 strains detected in 2005–2010 and 2011–2016. (DOC)

S1 Fig Phylogenetic analysis of the *env* region (HXB2: 6834–7289) of HIV-1 sequences in Mongolia. The evolutionary history was inferred using the neighbor-joining method with the Kimura 2-parameter method. The analysis involved 233 nucleotide sequences in the *env* region of HIV-1. MSM: men who have sex with men.
(TIF)

S2 Fig Mongolian B cluster illustrated by phylogenetic analysis of the *pol* region (HXB2: 2244–3308). The evolutionary history was inferred using the neighbor-joining method with the Kimura 2-parameter method. The Mongolian B cluster was composed of 60 Mongolian samples and 8 reference sequences. Bootstrap scores ≥90 are shown.
(TIF)

S3 Fig Mongolian B cluster illustrated by phylogenetic analysis of the *env* region (HXB2: 6834–7289). The evolutionary history was inferred using the neighbor-joining method with the Kimura 2-parameter method. The Mongolian B cluster was composed of 59 Mongolian samples. Bootstrap scores ≥90 are shown.
(TIF)

S4 Fig CRF51_01B cluster illustrated by phylogenetic analysis of the *pol* region (HXB2: 2244–3308). The evolutionary history was inferred using the neighbor-joining method with the Kimura 2-parameter method. CRF51_01B cluster was composed of 24 Mongolian samples and 6 reference sequences. Bootstrap scores ≥90 are shown.
(TIF)

S5 Fig CRF51_01B cluster illustrated by phylogenetic analysis of the *env* region (HXB2: 6834–7289). The evolutionary history was inferred using the neighbor-joining method with the Kimura 2-parameter method. CRF51_01B cluster was composed of 25 Mongolian samples and 5 reference sequences. Bootstrap scores ≥90 are shown.
(TIF)

S6 Fig Phylogenetic analysis of the near-full length HIV-1 genome (HXB2: 706–9531). The evolutionary history was inferred using the neighbor-joining method with the Kimura 2-parameter method. This tree was composed of 3 Mongolian samples, 5 reference sequences of CRF51_01B, and 26 other reference sequences. Bootstrap scores ≥90 are shown.
(TIF)

S7 Fig MCC tree of Bayesian MCMC analysis in the *env* region (HXB2: 6834–7289). Chronological phylogenetic relationship in the *env* region. The analysis involved 219 nucleotide sequences in the *env* regions (151 Mongolian samples and 68 reference sequences). Mongolian samples are indicated by red branches. Reference sequences are indicated by the black branches. The time scale is shown under the tree. (A) All sequences in the *env* region. Blue numbers show posterior probability. (B) Close up of CRF51_01B cluster in the *env* region. Blue circles: common ancestor. Data in square fields represent the median tMRCA and 95% highest posterior density interval. Numbers at the nodes represent posterior probability. MCC: maximum clade credibility. MCMC: Markov chain Monte Carlo. tMRCA: time to the most recent common ancestor.
(TIF)
