# Supplementary material for: The second molecular epidemiological study of HIV infection in Mongolia between 2010 and 2016
Source: PLoS One. 2017 Dec 15;12(12):e0189605. doi: 10.1371/journal.pone.0189605 (PMC5731755; doi:10.1371/journal.pone.0189605)
Supplement: S1 Table — (DOC) [file pone.0189605.s001.doc]

(A) All sequenced samples for the 2005-2016 period in Mongolia.

| Strain of HIV-1 | 2005-2010 | 2011-2016 | *p*^a^ |
| --- | --- | --- | --- |
| Dominantly circulating strains | 39 (69.6%) | 67 (69.1%) |  |
| Other strains | 17 (30.4%) | 30 (30.9%) |  |
| Total | 56 | 97 | 0.941 |

(B) Dominantly circulating strains of HIV-1 in Mongolia.

| Transmission route | 2005-2010 | 2011-2016 | *p*^b^ |
| --- | --- | --- | --- |
| MSM | 36 (92.3%) | 48 (71.6%) |  |
| Heterosexual males and females | 3 (7.7%) | 19 (28.4%) |  |
| Total | 39 | 67 | 0.013 |

(C) Other strains of HIV-1 in Mongolia.

| Transmission route | 2005-2010 | 2011-2016 | *p*^b^ |
| --- | --- | --- | --- |
| MSM | 6 (35.3%) | 8 (26.7%) |  |
| Heterosexual males and females | 11 (64.7%) | 22 (73.3%) |  |
| Total | 17 | 30 | 0.741 |

Dominantly circulating strains in Mongolia were Mongolian B, Korean B, and CRF51_01B.

^a^Pearson’s chi-square test. ^b^Fisher’s exact test.
