# Supplementary figures and images for: The second molecular epidemiological study of HIV infection in Mongolia between 2010 and 2016
Source: PLoS One. 2017 Dec 15;12(12):e0189605. doi: 10.1371/journal.pone.0189605 (PMC5731755; doi:10.1371/journal.pone.0189605)

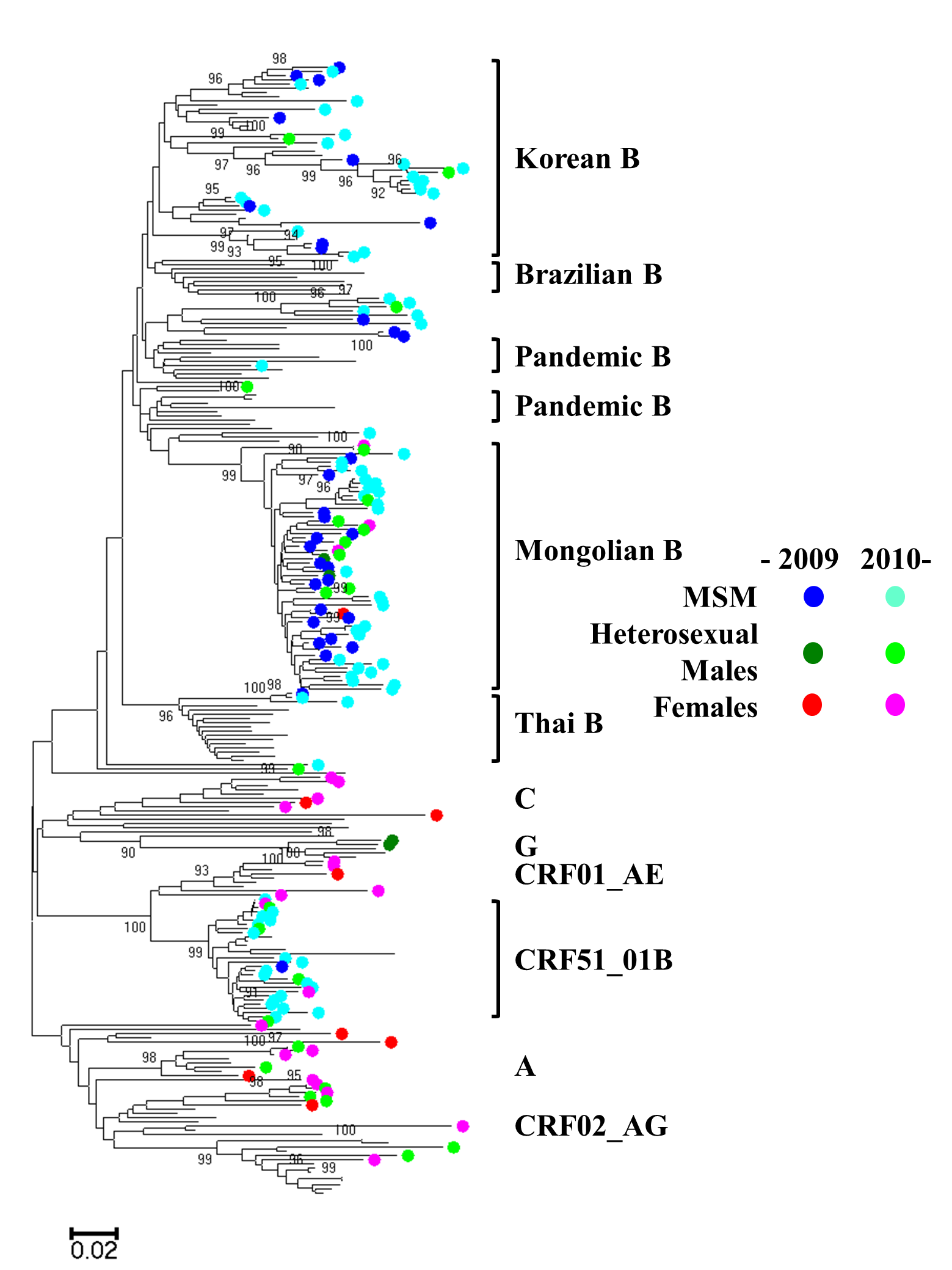

Supplement: S1 Fig — The evolutionary history was inferred using the neighbor-joining method with the Kimura 2-parameter method. The analysis involved 233 nucleotide sequences in the env region of HIV-1. MSM: men who have sex with men. (TIF) [file pone.0189605.s002.tif]

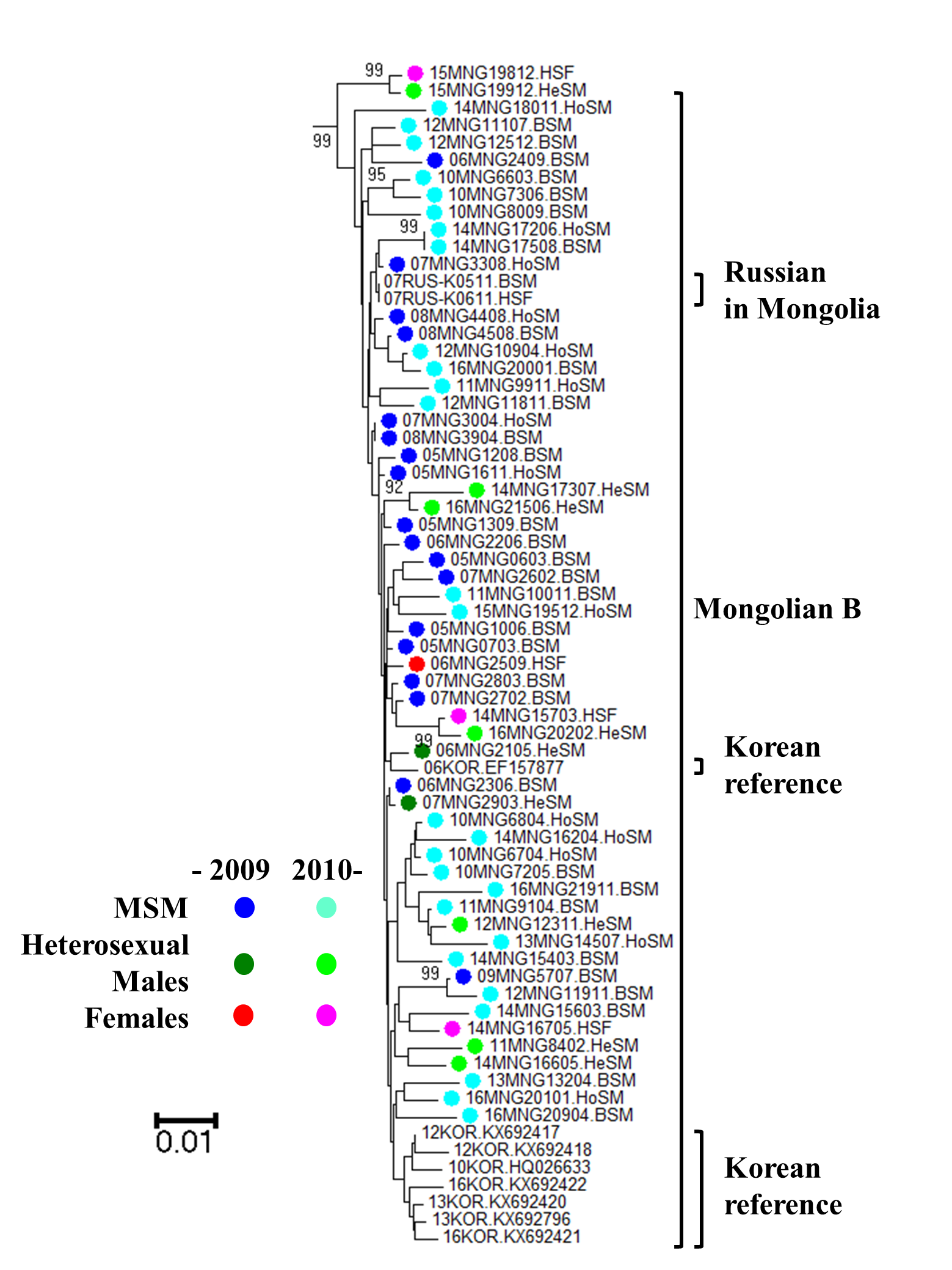

Supplement: S2 Fig — The evolutionary history was inferred using the neighbor-joining method with the Kimura 2-parameter method. The Mongolian B cluster was composed of 60 Mongolian samples and 8 reference sequences. Bootstrap scores ≥90 are shown. (TIF) [file pone.0189605.s003.tif]

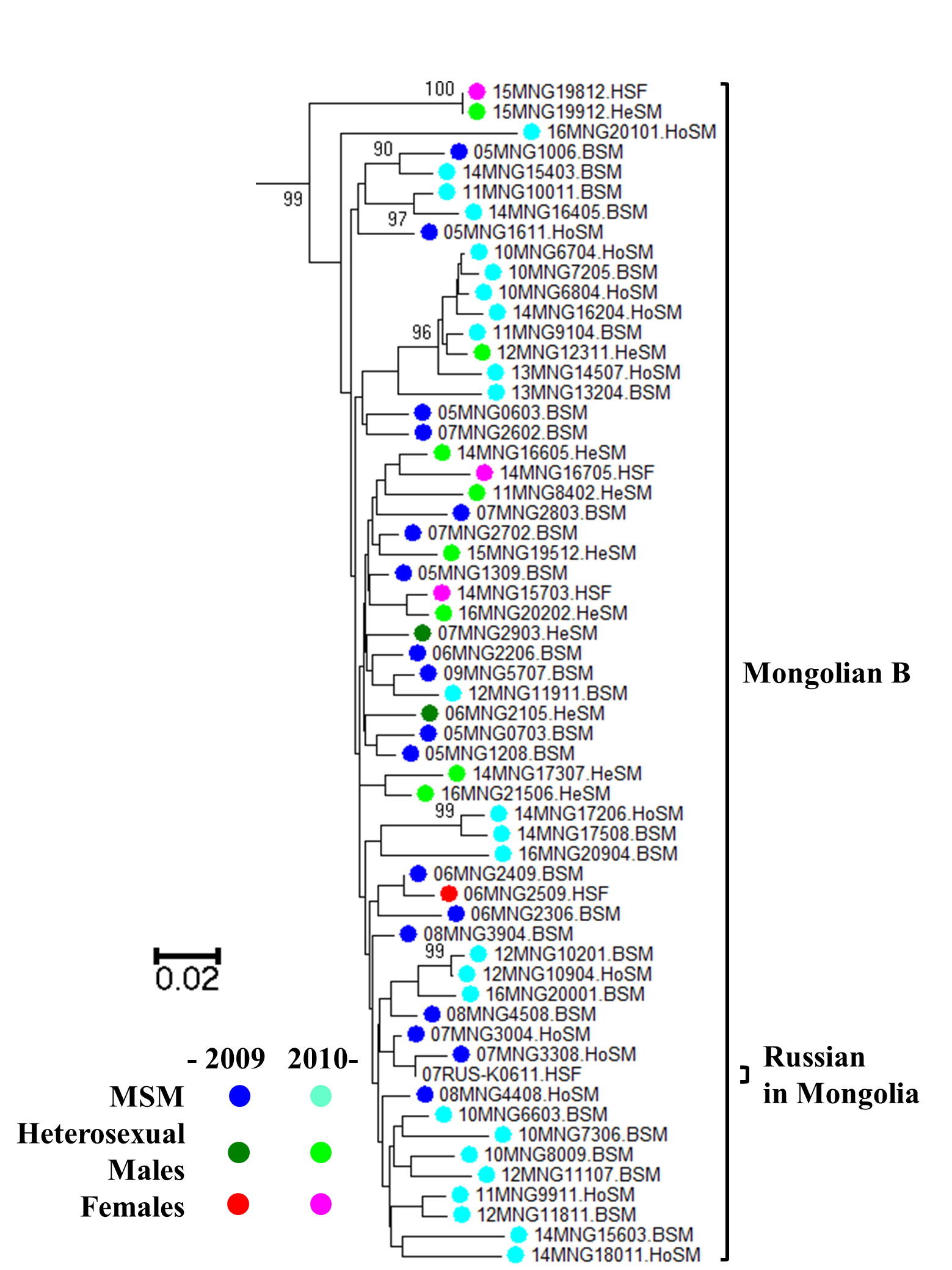

Supplement: S3 Fig — The evolutionary history was inferred using the neighbor-joining method with the Kimura 2-parameter method. The Mongolian B cluster was composed of 59 Mongolian samples. Bootstrap scores ≥90 are shown. (TIF) [file pone.0189605.s004.tif]

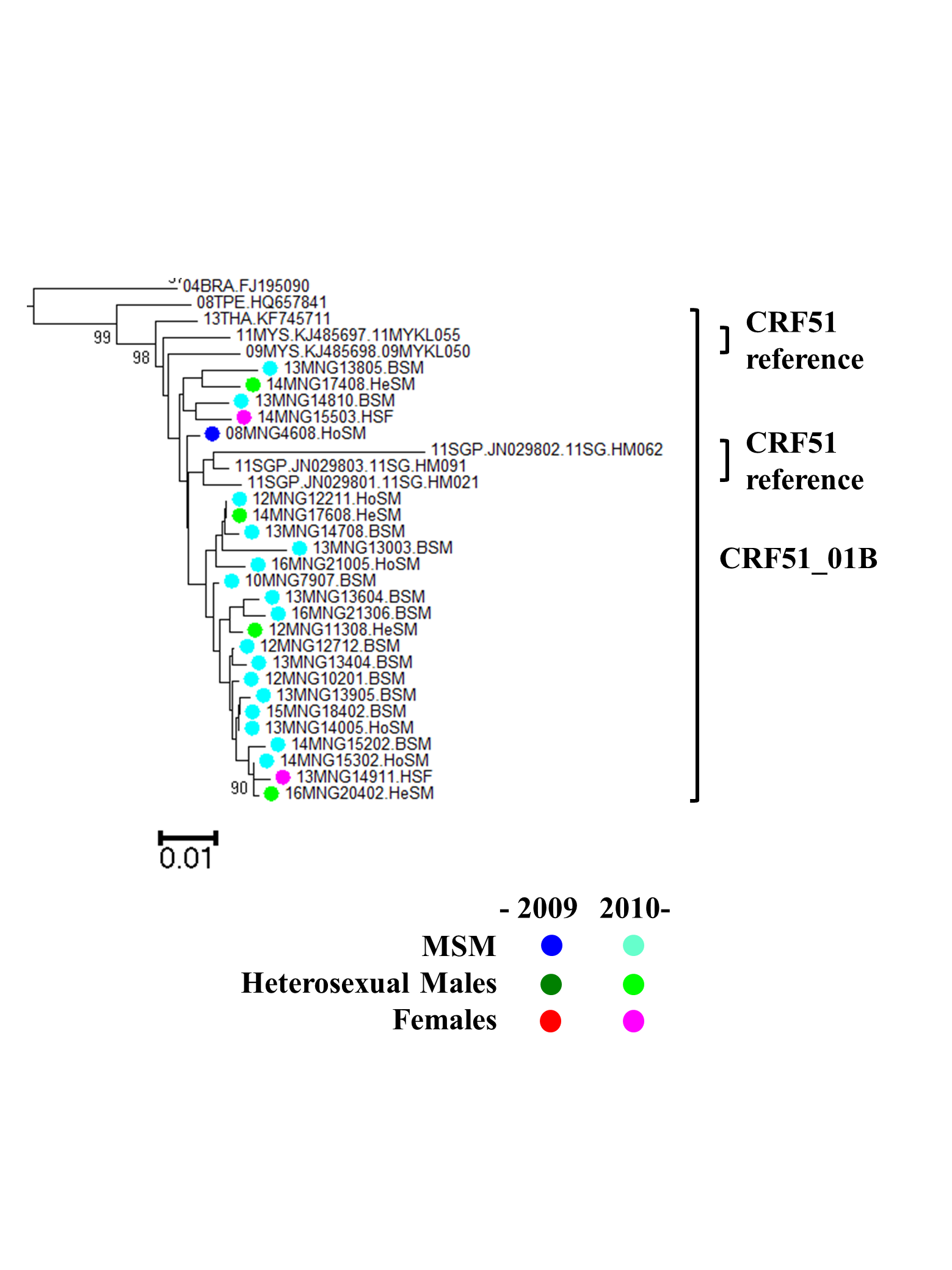

Supplement: S4 Fig — The evolutionary history was inferred using the neighbor-joining method with the Kimura 2-parameter method. CRF51_01B cluster was composed of 24 Mongolian samples and 6 reference sequences. Bootstrap scores ≥90 are shown. (TIF) [file pone.0189605.s005.tif]

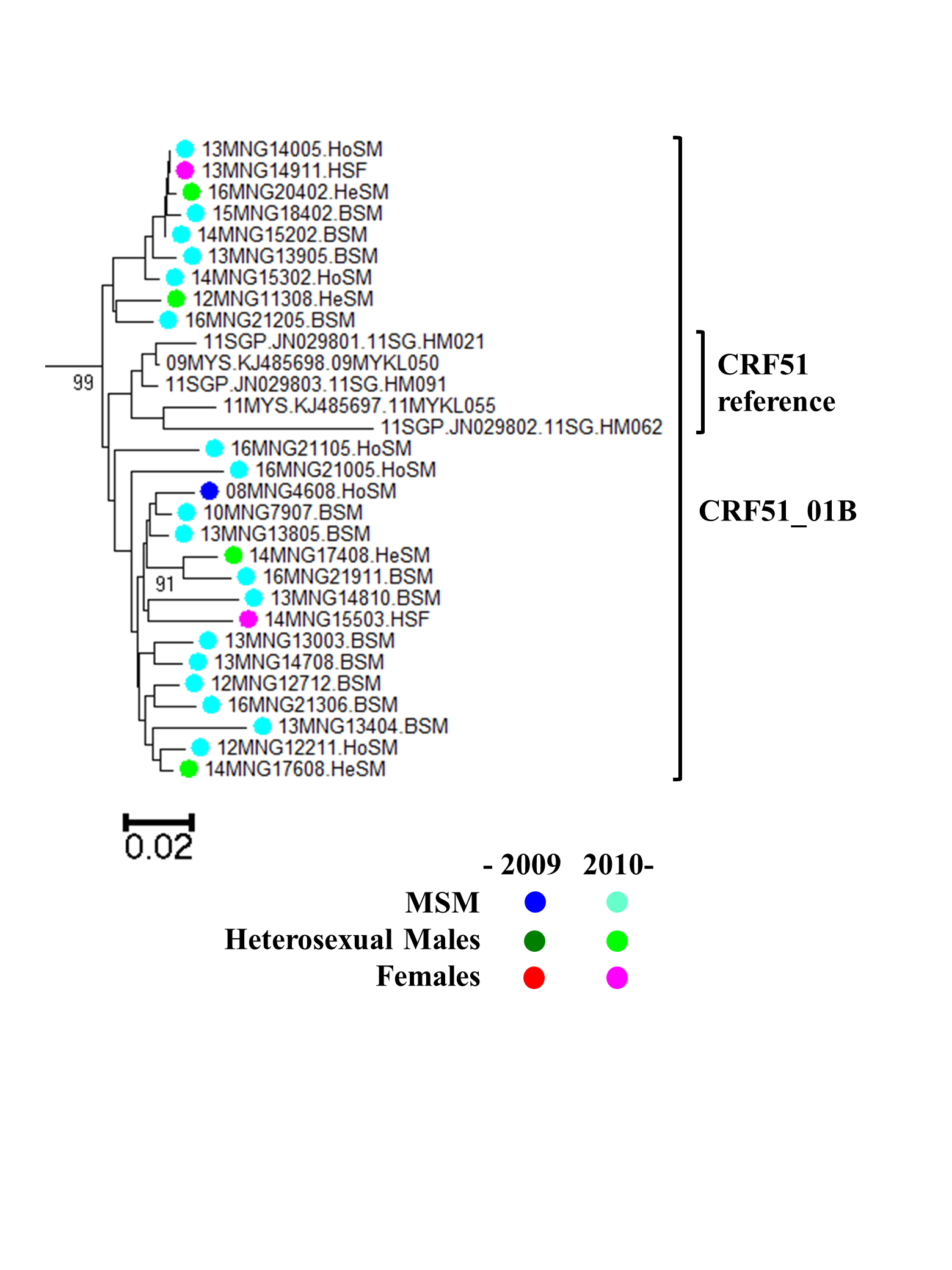

Supplement: S5 Fig — The evolutionary history was inferred using the neighbor-joining method with the Kimura 2-parameter method. CRF51_01B cluster was composed of 25 Mongolian samples and 5 reference sequences. Bootstrap scores ≥90 are shown. (TIF) [file pone.0189605.s006.tif]

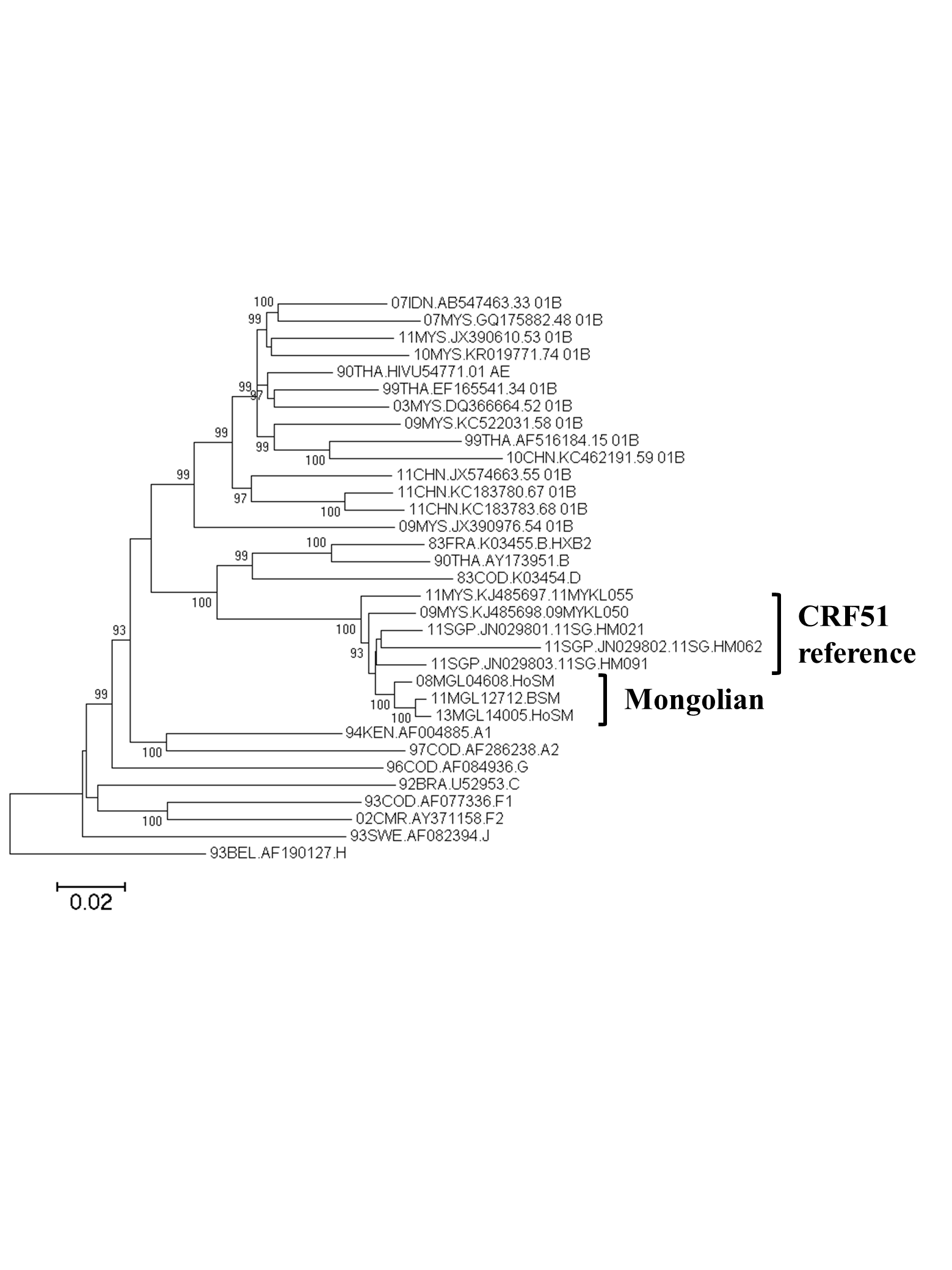

Supplement: S6 Fig — The evolutionary history was inferred using the neighbor-joining method with the Kimura 2-parameter method. This tree was composed of 3 Mongolian samples, 5 reference sequences of CRF51_01B, and 26 other reference sequences. Bootstrap scores ≥90 are shown. (TIF) [file pone.0189605.s007.tif]

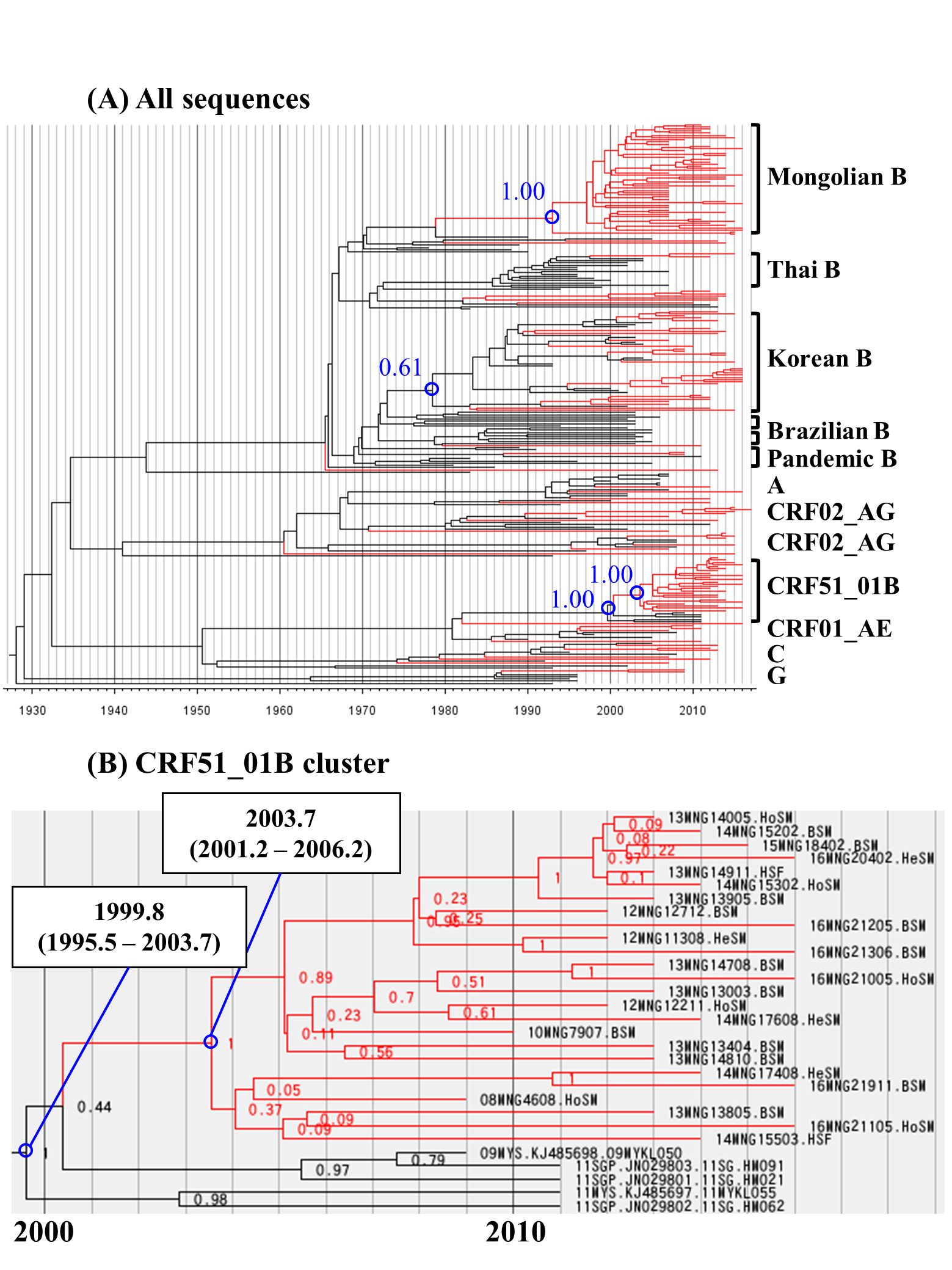

Supplement: S7 Fig — Chronological phylogenetic relationship in the env region. The analysis involved 219 nucleotide sequences in the env regions (151 Mongolian samples and 68 reference sequences). Mongolian samples are indicated by red branches. Reference sequences are indicated by the black branches. The time scale is shown under the tree. (A) All sequences in the env region. Blue numbers show posterior probability. (B) Close up of CRF51_01B cluster in the env region. Blue circles: common ancestor. Data in square fields represent the median tMRCA and 95% highest posterior density interval. Numbers at the nodes represent posterior probability. MCC: maximum clade credibility. MCMC: Markov chain Monte Carlo. tMRCA: time to the most recent common ancestor. (TIF) [file pone.0189605.s008.tif]
